# Efficient dehydration and recovery of ionic liquid after lignocellulosic processing using pervaporation

**DOI:** 10.1186/s13068-017-0842-9

**Published:** 2017-06-15

**Authors:** Jian Sun, Jian Shi, N. V. S. N. Murthy Konda, Dan Campos, Dajiang Liu, Stuart Nemser, Julia Shamshina, Tanmoy Dutta, Paula Berton, Gabriela Gurau, Robin D. Rogers, Blake A. Simmons, Seema Singh

**Affiliations:** 1 Deconstruction Division, Joint BioEnergy Institute, Emeryville, CA 94608 USA; 2 Biological and Engineering Sciences Center, Sandia National Laboratories, Livermore, CA 94551 USA; 3 Biosystems and Agricultural Engineering, University of Kentucky, Lexington, KY 40546 USA; 4 Biological Systems and Engineering Division, Lawrence Berkeley National Laboratory, Berkeley, CA 94720 USA; 5 Compact Membrane Systems Inc, Newport, DE 19804 USA; 6 Department of Chemistry, The University of Alabama, Tuscaloosa, AL 35487 USA; 7 Department of Chemistry, McGill University, 801 Sherbrooke St. West, Montreal, QC H3A 0B8 Canada; 8 525 Solutions, Inc., Tuscaloosa, AL 35401 USA

**Keywords:** Biomass pretreatment, Ionic liquid, Saccharification, Biofuels, Recycle, Pervaporation

## Abstract

**Background:**

Biomass pretreatment using certain ionic liquids (ILs) is very efficient, generally producing a substrate that is amenable to saccharification with fermentable sugar yields approaching theoretical limits. Although promising, several challenges must be addressed before an IL pretreatment technology can become commercially viable. One of the most significant challenges is the affordable and scalable recovery and recycle of the IL itself. Pervaporation (PV) is a highly selective and scalable membrane separation process for quantitatively recovering volatile solutes or solvents directly from non-volatile solvents that could prove more versatile for IL dehydration.

**Results:**

We evaluated a commercially available PV system for IL dehydration and recycling as part of an integrated IL pretreatment process using 1-ethyl-3-methylimidazolium acetate ([C_2_C_1_Im][OAc]) that has been proven to be very effective as a biomass pretreatment solvent. Separation factors as high as 1500 were observed. We demonstrate that >99.9 wt% [C_2_C_1_Im][OAc] can be recovered from aqueous solution (≤20 wt% IL) and recycled five times. A preliminary technoeconomic analysis validated the promising role of PV in improving overall biorefinery process economics, especially in the case where other IL recovery technologies might lead to significant losses.

**Conclusions:**

These findings establish the foundation for further development of PV as an effective method of recovering and recycling ILs using a commercially viable process technology.

**Electronic supplementary material:**

The online version of this article (doi:10.1186/s13068-017-0842-9) contains supplementary material, which is available to authorized users.

## Background

**Fig. 1:**
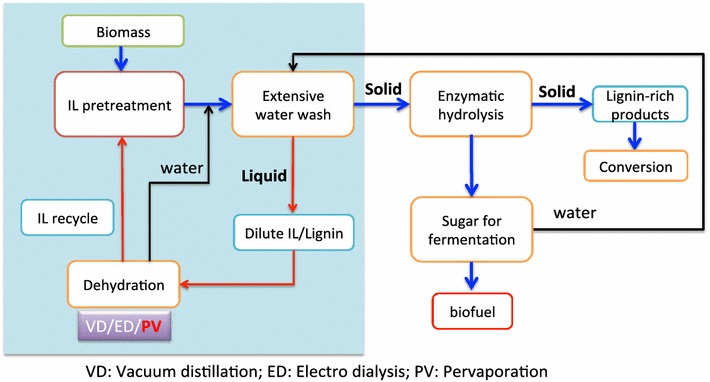
Simplified process flow diagram for the potential ionic liquid-based biorefinery and performance evaluation of pervaporation, ED and VD in one of the water-wash process scenarios

Certain ionic liquids (ILs), e.g., 1-ethyl-3-methylimidazolium acetate ([C_2_C_1_Im][OAc]) and 1-butyl-3-methylimidazolium chloride ([C_4_C_1_Im]C_l_), have been demonstrated to be very effective at pretreating a wide range of lignocellulosic biomass feedstocks that are capable of generating very high yields of fermentable sugars suitable for biofuel production via fermentation (Fig. 1) [1–3], and thus have been widely used recently [4]. Although promising, the costs associated with this pretreatment technology are still considered by many to be prohibitive. One of the challenges is the intrinsic cost of the IL itself and the need for effective means of recovery and recycle [4–6]. For instance, at an IL recovery of 99.5%, the cost contribution due to the lost IL could be in the range of $0.3 gal^−1^ (at 30% solids loading with $2 kg^−1^ IL) to $5.3 gal^−1^ (at 10% solids loading with $10 kg^−1^ IL) even if the sugar yields are high [7]. This emphasizes the need for technologies that can minimize IL losses during recycle, thereby facilitating high IL recoveries (>99%). IL dehydration is an important step due to the need for water washing of pretreated biomass to reduce the inhibitory effect of certain ILs, including [C_2_C_1_Im][OAc], to enzymes and microbes during enzymatic hydrolysis and fermentation [8–11]. With the use of large quantities of water in this step, the solids are precipitated/separated and the IL is simultaneously recovered into the aqueous stream. It is therefore imperative to develop affordable and robust dehydration technologies that can recover ILs from aqueous solutions while minimizing any IL losses during the recovery process.

**Fig. 2:**
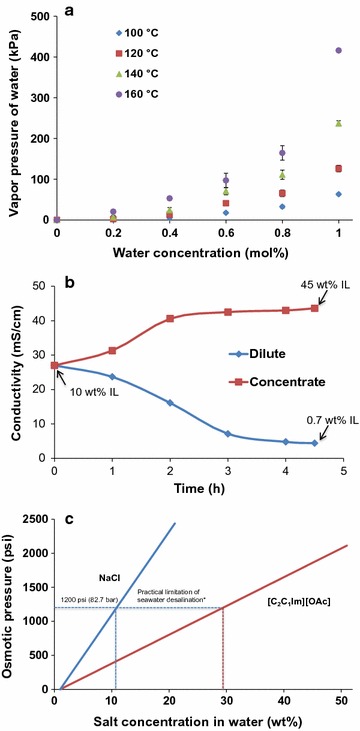
Challenges related to high IL dehydration in current IL separation methods: **a** distillation, **b** electrodialysis and **c** reverse osmosis. Detailed information on Fig. 2b is described in Additional file 1: Figure S7; data in Fig. 2c on upper limit of osmotic pressure for an industrial setting were obtained from Reference [20]

Separation technologies such as evaporation/distillation, electrodialysis (ED), reverse osmosis (RO) have been investigated for concentrating ILs [12–15]. As known distillation is considered as the simplest method for removal of volatile solvents and solutes from ILs, and the volatile compounds can be distilled by vacuum evaporation, wiped film evaporation, column distillation, and molecular distillation [12]. However, distillation suffers from high energy consumption and low separation selectivity [16, 17]. In the case of an IL/H_2_O mixture, high temperature and vacuum are needed to break the strong IL–H_2_O interactions that are dependent on the amount of water present and are stronger at lower water concentrations [18]. Water molecules can form hydrogen bonds with anions in imidazolium-based ILs and at low water concentrations water molecules prefer to form complexes mostly with anions rather than with other water molecules [19]. The measured vapor pressures of the binary [C_2_C_1_Im][OAc]/H_2_O system in the temperature range of 100–160 °C (Fig. 2a) show that the binary mixture has a negative deviation from Raoult’s Law, confirming the strong interactions between [C_2_C_1_Im][OAc] and H2O. Consistent with the observation from a previous work [20], Fig. 2a also illustrates a ‘boiling-point elevation’ (above 100 °C) of water when [C_2_C_1_Im][OAc] is added. In addition, there may be significant IL losses associated due to physical carryover in a typical distillation setup where there is no physical barrier to prevent any carryover losses. It is therefore challenging to achieve both highly concentrated ILs and quantitative IL recovery by distillation [21].

ED is a membrane-based process that has been applied for desalination of seawater and brackish water [22], however, only ~85% recovery of [C_4_C_1_Im]Cl could be realized with this technique [13, 23]. Furthermore, ED suffers from low efficiency limits in terms of final IL concentration achieved (Fig. 2b) due to conductivity/viscosity constraints, fouling, and relatively short membrane lifetime. Lastly, RO, which involves the application of pressure to the liquid–water feed, forcing smaller water molecules through a membrane, would require significant energy due to the need for high pressure at higher IL concentrations [24]. In addition, the very high osmotic pressure required to concentrate the dilute IL stream to a pretreatment relevant concentration makes this technology impractical in reality due to a practical limitation of 1200 psi (Fig. 2c) [25, 26]. Thus, there is a clear need for an efficient, affordable, and scalable method to dehydrate IL to relatively high concentration (i.e., with effective water content of <10 wt%), while maintaining high IL recovery (>99%), from aqueous mixtures after pretreatment.

Membrane-based pervaporation (PV) is emerging as an alternative to these technologies and has the potential to reduce energy usage and operating costs [27, 28]. In PV, a fraction of the liquid feed can be selectively evaporated under moderate conditions via the physical–chemical interactions between the membrane material and the permeating molecules, not the relative volatility as in distillation, thus significantly reducing the amount of energy required relative to technologies in which the entire stream is evaporated [28–30]. PV can be applied in biotechnology to concentrate heat-, stress-, and/or chemical-sensitive biochemicals [27, 31–33]. PV appears to be amenable to effective water/IL, volatile organic solvent/IL or organic solvent/water separations [27, 29, 30, 34, 35]. In the context of IL dehydration, as shown in this work, the PV membrane exhibits excellent resistance to IL permeation, thereby minimizing IL loss while, at the same time, achieving high levels of dehydration to recover IL in its concentrated form (~99 wt% IL). In this work, we used a commercially available PV unit that utilizes a perfluorinated membrane, obtained from Compact Membrane Systems Inc. (CMS), to evaluate and assess the potential of PV for IL dehydration in a relevant lignocellulosic processing environment. We first establish the basic performance metrics for the dehydration of a water-IL binary mixture, and then demonstrated that >99.9 wt% [C_2_C_1_Im][OAc] IL could be recovered from aqueous solution by PV and reused at least five times in a biomass pretreatment process. A preliminary technoeconomic analysis indicates that PV is a promising technology for the efficient dehydration and recycle of ILs primarily due to its ability to minimize (or avoid) IL losses.

## Results and discussion

### Pervaporation setup

**Fig. 3:**
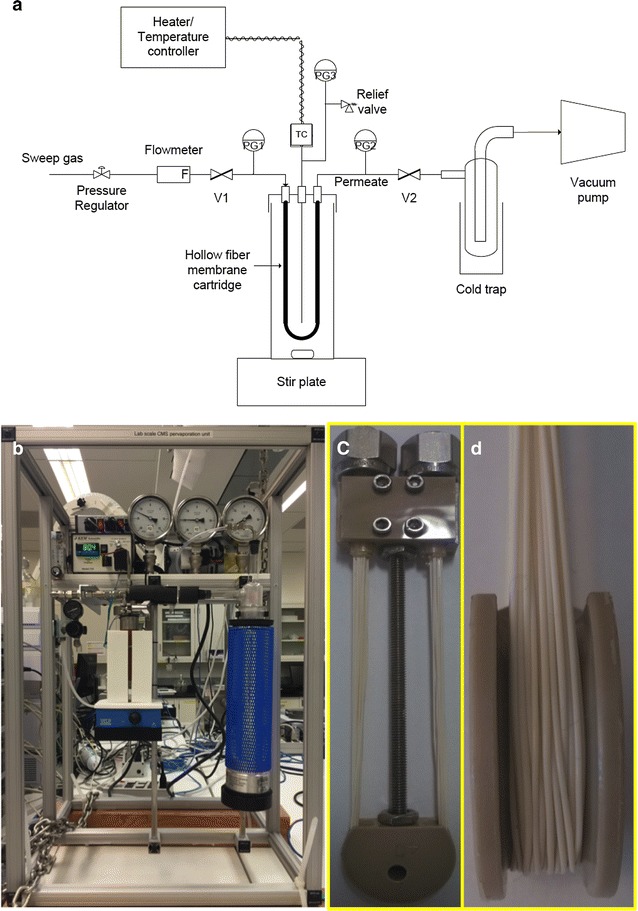
Schematic diagram **a** and picture **b** of the lab-scale apparatus used for PV, and employed hollow fiber membranes (**c** and **d**)

A schematic diagram of the system used to carry out the pervaporation (PV) experiments is shown in Fig. 3a. In a lab-scale PV unit (Fig. 3b), the membrane is made of a thin dense layer of a fluoropolymer coated on a hollow fiber microporous support. The lab permeation module used consisted of about 16 fibers (microtubes) about 8’ long (Fig. 3c, d) and were directly immersed into the feed solution that is stored in a 125 mL stainless steel vessel. The effective membrane surface area used in the membrane stability experiments conducted at the Joint BioEnergy Institute (JBEI) is ~90 cm^2^, while it is 60 cm^2^ in the experiments conducted at Compact Membrane System (CMS). The combination of feed mass balance and conductivity measurement of the sample was used as a quick determination of the IL concentration. During PV process, feed is allowed to flow along one side of the membrane and a fraction of the feed (permeate) passes through the membrane and enters the vapor phase on the opposite side of the membrane. The “vapor phase” side of the membrane is kept under a vacuum or it is purged with a stream of inert carrier gas. The permeate is finally collected in the liquid state after condensation.

Conductivity measurements [36, 37] were performed at room temperature (~20–23 °C) to track the amount of [C_2_C_1_Im][OAc] present both in the permeate and feed before, during, and after dehydration. It was found that conductivity increases from the value of 7.6 mS cm^−1^ for ‘pure’ [C_2_C_1_Im][OAc], presents a maximal value of 39 mS cm^−1^ at a 30 wt% IL concentration, and then decreases to around 0.02 mS cm^−1^ with further increase of water concentration to over 99.9 wt% water (Additional file 1: Figure S1a). Derived from the experimental data and a previous report [36], possible aggregation of the [C_2_C_1_Im][OAc] in aqueous solutions is attributed as the main reason that a low conductivity in the IL-rich range is observed. However, a linear plot of the conductivity with the concentration of IL was found in the low concentration range (Additional file 1: Figure S1b), which was used to detect the IL loss in the permeate after dehydration. IL recovery is defined as the percent of the initial mass of IL that can be recovered by dehydration. During dehydration, the IL degradation under the PV conditions (50–100 °C) was negligible (Additional file 1: Figure S2). The initial conductivity value of IL feed solution (i.e., 20 wt%) is 36.0 ± 0.1 mS cm^−1^.

Operating parameters, i.e., temperature, time, and feed mass; and membrane parameters, i.e., permeation flux, IL/H_2_O separation factor, water permeability, and stability, are here studied to understand and improve the PV performance for the dehydration of the [C_2_C_1_Im][OAc]/H_2_O mixture.

### Effect of operating parameters on PV performance

In order to determine the impact of operating temperature, time, and initial feed mass on the performance of the PV unit for IL dehydration and recovery, a series of experiments were carried out where the initial concentration of [C_2_C_1_Im][OAc] in feed was at around 20 wt%. Temperature plays an important role on the rate and extent of dehydration, as water vapor pressure is a function of the temperature. Moreover, the previous work [20] has demonstrated that the presence of ILs can significantly increase the boiling point of water, possibly because of the strong interactions between IL and H_2_O.

**Fig. 4:**
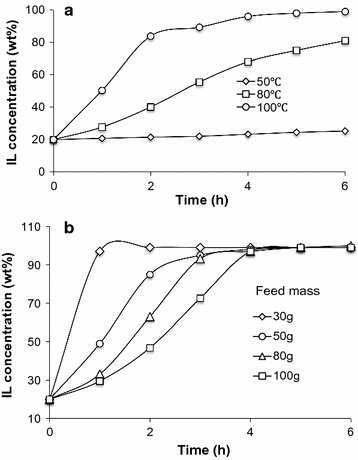
Effects of temperature (**a**) and initial IL feed mass (**b**) on [C_2_C_1_Im][OAc] concentration as a function of time (@ 100 °C)

There was almost no dehydration observed at 50 °C, even after 6 h of operation (Fig. 4a). When the temperature was elevated to 80 °C, a significant change in [C_2_C_1_Im][OAc] concentration was observed. During the first 4 h of operation, a linear dehydration curve was established, indicating a constant dehydration rate. After 4 h, the dehydration rate started to decrease. After 6 h of operation, the [C_2_C_1_Im][OAc] concentration reached a maximum value of ~80 wt%, although the slope of dehydration profile remained slightly positive. As the temperature was increased to 100 °C, a greater dehydration rate was observed within the first 2 h of operation, and the concentration of [C_2_C_1_Im][OAc] reached ~80 wt%, and the dehydration curve reached a maximum value of ca. 99 wt% [C_2_C_1_Im][OAc] after 4 h of operation. Thus, 100 °C was used in this study for the separation of [C_2_C_1_Im][OAc]/H_2_O system in order to obtain high dehydration rate. Compared to evaporation that is based on the same driving force (i.e., water vapor pressure), the PV membrane provides a barrier, and thus minimizes losses of [C_2_C_1_Im][OAc].

Data indicate that lower initial feed mass renders faster water removal from [C_2_C_1_Im][OAc] (Fig. 4b). For example, when the initial mass was relatively low (e.g., 30 g), only 1–2 h was needed to achieve near total dehydration of the [C_2_C_1_Im][OAc] (99 wt%). When the initial mass was increased up to 50, 80, or 100 g, it required 4, 5, and 6 h, respectively, to reach the near total dehydration of the IL. Noting that the above observations reflect a dynamic change of mass loading and membrane area in contact, water flux was calculated to obtain a better understanding of PV efficiency.

### Membrane relevant parameters and recommended operational protocol

The average water flux was calculated using the following equation.

1 $$ \eta = m_{{{\text{H}}_{ 2} {\text{O}}}} /t/A \, $$

where *η* is the average water flux, *m*H_2_O is the mass of permeated water, *t* is the separation time, and *A* is the membrane area in contact with the liquid.

**Fig. 5:**
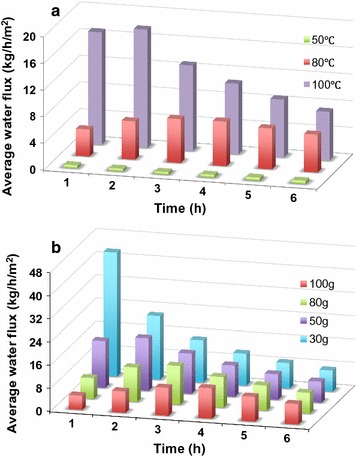
Effects of temperature (**a** initial feed mass fixed at 50 g) and initial I L feed mass (**b** temperature fixed at 100 °C) on water flux of the PV

**Fig. 6:**
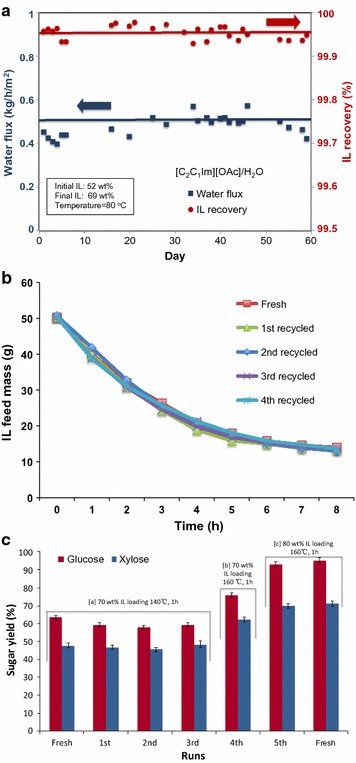
Stability of the pervaporation membrane as a function of time and usage (**a** and **b**), and performance of recycled IL for biomass pretreatment (**c**). Biomass loading in pretreatment step is fixed at 10 wt%

The calculated average water fluxes (Fig. 5) as a function of temperature and initial IL feed mass are in agreement with the experimental results presented in Fig. 4a, b, respectively. At 50 °C, the water flux was almost constant at 0.4–0.5 kg h^−1^ m^−2^ for the entire run (Fig. 5a). A significant improvement in the average water flux was observed when the temperature was increased to 80 °C (4.1–6.7 kg h^−1^ m^−2^) or 100 °C (7–18 kg h^−1^ m^−2^). Initial average water flux was observed to decrease with increase in initial feed mass (Fig. 5b). In the case of 30 g initial mass, the maximum average water flux reached to 42.8 kg h^−1^ m^−2^. With 100 g initial mass of IL solution, the initial average water flux was reduced to 5 kg h^−1^ m^−2^. In this work, the increased or reduced flux of water with varying time can be ascribed to a comprehensive function of the time and total membrane area in contact with the feed solution. It is important to note that the water flux can be constant in a continuous PV process (Fig. 6a), where the feed is in contact with the entire membrane area during the entire process. In a continuous process, the membrane module is fed continuously; the commercial module is designed so that there is very good mixing of the liquid inside the module, with negligible stagnant zones or bypass, so that fresh feed thoroughly contacts the entire membrane area.

Separation factor is a commonly used metric to evaluate the PV membrane separation efficiency. The H_2_O–IL separation factor is defined by the following Eq. (2).

2 $$ \text{SF}_{{w - \text{IL}}} = \frac{{\frac{{x_{w,P} }}{{x_{{\text{IL},P}} }}}}{{\frac{{x_{w,F} }}{{x_{{\text{IL},F}} }}}}, $$

where SF_*w*_-IL is H_2_O–IL separation factor, *X*_*w*_,_*P*_ is mass fraction of H_2_O in the permeate, X_IL_,_*P*_ is mass fraction of IL in the permeate, X_*w*_,_*F*_ is mass fraction of H_2_O in the feed, X_*IL*_,_*F*_ is mass fraction of IL in the feed. Based on the tests done at CMS, the calculated average water-IL separation factor is around 1500, indicative of high IL recoveries.

The water permeability is defined as the product of the water permeance and the membrane thickness. The water permeance is calculated as the ratio of the water flux to the water driving force across the membrane. The water driving force is the difference between the partial pressure of water on the feed side and the partial pressure of water on the permeate side. Based on the experimental data from CMS, the calculated average water permeability of the CMS membrane is about 2200 barrer (1 Barrer is equivalent to 3.34 × 10^−16^ mol Pa^−1^ s^−1^ m^−1^).

An important observation was that if the membrane was left impregnated with the IL at the end of an experiment, it confounded the results of the subsequent experiment. Additional file 1: Figure S3a shows that if membrane is not properly rinsed between experiments, the water content did not drop below the threshold value of 16.5 ± 0.2 wt% water, independent of initial water content. Since the data collected for this study are based on batch experiments, it was important to remove residual IL from the membrane before the next experiment, so there is no cross-contamination between the tests. Our investigation provided insight that a water rinse of the membrane is sufficient to remove most of the IL that impregnated the membrane. This protocol is effective because the IL is highly soluble in water. Thus, after a water rinse, no IL was left on the membrane. The results show that when the proper care is taken of the membrane, 5 h of IL treatment resulted in water content as low as 4.7 ± 0.2 wt% (Additional file 1: Figure S3b).

### Membrane stability and IL recycle performance

The investigations of membrane stability were conducted separately at JBEI and CMS using the same lab-scale (125 mL) PV unit but with different membrane surface areas. The performance and the stability of the membrane in terms of water flux and IL recovery in 60 cycles of the [C_2_C_1_Im][OAc]/H_2_O mixture was investigated at CMS, where the effective membrane surface area was around 60 cm^2^ (Fig. 6a).

Results show that the water flux stays fairly constant and averages about 0.47 kg h−1 m−2. Also, the IL recovery stayed at the very high value of about 99.95% (i.e., only ~0.05% IL is lost in the permeate). The test was run daily for 60 days with [C_2_C_1_Im][OAc] at 80 °C using the same membrane. Each day, the test was run in the lab-scale PV unit with 50 g of IL containing about 52 wt% water. At the end of each test, which lasted 5 h, the final water concentration was about 31 wt%. The next day, the permeate was added back to the dehydrated IL and a consistent feed solution was used for the next run. In this work, conductivity of the feed solution was measured before the investigation of membrane stability. By maintaining a consistent feed solution of similar conductivity values, consistency was maintained during the study. This step was reiterated for 60 dehydration cycles using the same membrane.

In the case of IL recycle performance evaluation, in order to eliminate fouling of the membrane by contaminants present after pretreatment, ultrafiltration (UF) was used with a 30 kD polysulfone UF ER membrane (Sterlitech, Co., Lot# XDAXAC) (operation conditions: 1 MPa N_2_, 20 °C, overnight) to remove the majority of the soluble lignin and other solutes present in the aqueous IL solution (Additional file 1: Figure S4a).

We performed SEM and FTIR characterizations on the new and used polysulfone UF ER membranes. SEM results suggest that the membrane retained its integrity and no physical damage was observed after five runs (Additional file 1: Figure S4b), and the FTIR results confirm that lignin residues can be easily washed off using water and that the UF membrane is chemically stable (Additional file 1: Figure S4c). Thereafter, the dehydration efficiency of the PV system was evaluated under the same conditions (50 mL feed mass, 100 °C) (Fig. 6b), with no observable changes after 4 cycles. The IL-rich phase recovered after PV, which contained below 0.01 wt% amounts of xylan and lignin, was reused directly for biomass pretreatment.

To determine the performance of recycled [C_2_C_1_Im][OAc], three IL recycles were conducted under relatively moderate pretreatment conditions (140 °C, 70 wt% IL loading) (Fig. 6c, panel a). When compared to the fresh IL, the recycled IL performed well and yielded comparable sugar release profiles after saccharification. With increasing temperature (from 140 to 160 °C) and IL loading (from 70 to 80 wt%) in the pretreatment, a further increase of sugar yields can be achieved in the 4th and 5th IL recycles (Fig. 6c, panels b, c), which demonstrated that IL could be reused for five times without significant IL loss or negative impact in terms of pretreatment efficiency. A preliminary mass balance for [C_2_C_1_Im][OAc] and water was performed using an initial 100 g basis of the raw mixed feedstock (Additional file 1: Figure S5). Greater than 99.9% IL could be repeatedly recovered from the aqueous solution by PV. The comparison of 1H-NMR spectra obtained from fresh and the recycled IL indicates that the structure of IL was stable without significant change (Additional file 1: Figure S6a, b). Also, there is no visible loss of [C_2_C_1_Im][OAc] in the permeate based on 1H-NMR spectra (Additional file 1: Figure S6c).

### Comparison of different separation methods

The technical comparisons of PV with ED and VD for IL dehydration were evaluated on a stream of IL-water mixture generated from a mixed feedstock pretreatment are summarized in Table 1.

**Table 1:** Technical comparisons of different methods for the dehydration of [C_2_C_1_Im][OAc]/H_2_O system

Entry	Item	IL:H_2_O (w/w)	t/P (h/kPa)	Final IL concentration (wt%) (°C)	IL loss (wt%)
1	PV	20:80	4/~12	>99 (@100)	0.02–0.04
2^a^	ED	10:90	4/−	45 (@20)	7.0
3	VD	20:80	4/10	90 (@100)	0.1
4	VD	53:47	13.8	69 (@80)	0.15
5^b^	PV	53:47	13.8	69 (@80)	0.03

Initial feed mass (50 g)

*PV* pervaporation, *VD* vacuum distillation, *ED* electrodialysis

^a^Pressure was not detected

^b^2.5 kg initial feed mass in a 3 L scale-up PV apparatus at CMS

The results confirm that PV is the most efficient method among the three approaches investigated (entries 1–3) and both >99 wt% IL concentration and negligible IL loss (0.02 wt%) can be achieved reproducibly. The negligible IL loss observed in the experiments indicates a very large membrane selectivity of H_2_O to IL that is the benefit provided by PV process. The loss of IL in the PV process was mainly caused by the negligible permeation from the membrane, and detected by the conductivity of water dehydrated by PV. In the case of VD (entries 3 and 4), the loss of IL is relatively higher (0.1–0.15 wt%) than that of PV, which is caused by the liquid entrainment during the vigorous vacuum evaporation process. In addition, only 90 wt% IL concentration can be reached under the same conditions. In this work, the comparisons of PV with VD (entry 1 vs. 3; and entry 4 vs. 5) were conducted under the same temperature and [C_2_C_1_Im][OAc] concentration.

Results obtained from bench-top ED apparatus in our lab (Additional file 1: Figure S7) indicate that only ~45 wt% final [C_2_C_1_Im][OAc] concentration was obtained after 4.5 h dehydration using ED starting at 10 wt% [C_2_C_1_Im][OAc] (Table 1; Fig. 2b). Compared to PV, the IL loss in ED is fairly high (~7.0 wt%) (Table 1, entries 1 and 2) and thus is unacceptable to meet the needs of >99% high IL recovery. The low efficiency and recovery limits of ED in terms of final [C_2_C_1_Im][OAc] concentration, are possibly caused by the limiting current density [38], conductivity and viscosity constraints, membrane fouling and relatively short lifespan of the ED membrane. In addition to being less efficient in terms of IL recovery and level of dehydration, the energy intensity of ED process is likely to be high with reported specific energy consumption ranging from 514 g kWh^−1^ [39] to 1350 g kWh^−1^ [13].

### Technoeconomic analysis

**Fig. 7:**
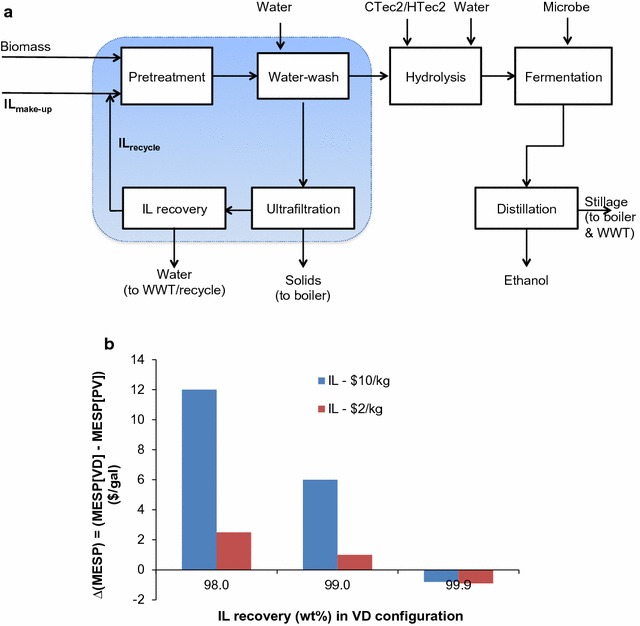
Integrated biorefinery model (**a**) and impact of IL recovery on minimum ethanol selling price (**b**)

In order to understand the impact of IL recovery on the overall biorefinery economics, a preliminary technoeconomic analysis (TEA) was conducted. To facilitate the TEA, an integrated biorefinery model (Fig. 7a) was built, which represents a mature industrial scale facility (i.e., Nth plant) that is capable of processing 2000 MT/day dry biomass. Details on the biorefinery configuration are discussed in the Experimental section and key process specifications are also provided (Additional file 1: Table S1). Essentially, the biorefinery process configuration is based on the design proposed by National Renewable Energy Laboratory (NREL) [40], except the pretreatment (including IL recovery/recycle) configuration. The overall process consists of multiple unit operations including IL pretreatment (and IL recovery), hydrolysis, fermentation, product recovery, wastewater treatment (WWT), and on-site co-generation.

Using this integrated biorefinery model to evaluate the economics of VD and PV systems for IL recovery (from the aqueous IL stream from the water-washing step), two different configurations were constructed (Additional file 1: Figure S8). The first configuration (‘PV/hybrid’) is a hybrid configuration that employs an initial feed concentration (from around 85 to 60 wt% water), followed by PV to further reduce water content to 10 wt%. In the second configuration (‘VD’), only VD is employed. Given the energy intensive nature of this IL recovery system, in both the configurations, process intensification is considered thus utilizing ‘multi-effect’ vacuum systems, which are commonplace in other facets of industry (e.g., desalination) to improve overall energy efficiency. To this end, in the PV/hybrid configuration, similar to a multi-effect evaporation system proposed by NREL [41], a multi-effect VD (MEVD) with three effects/stages is considered, followed by a PV membrane. In the case of the VD configuration, MEVD system with an additional stage (i.e., 4 effects in total) is considered. In addition, whenever possible, heat integration between different sections in the biorefinery (e.g., between product recovery and IL recovery sections) is employed—in both configurations—to further reduce energy needs. Latent heating needs in both configurations are supplied by drawing steam from the appropriate turbine section (low pressure or medium pressure, as required). Both of these configurations are discussed in the Experimental section in more detail.

The PV/hybrid and VD configurations are fundamentally different. For instance, the benefit of the PV/hybrid configuration lies in the fact that it combines the scalability and energy efficiency of the MEVD systems (for initial aqueous IL concentration), together with the near-complete IL recovery potential of PV system in the subsequent dehydration step at 100 °C. In addition, due to significant removal of water during the initial concentration step, relatively much smaller PV system would be required downstream for subsequent dehydration. The VD configuration is relatively simpler (as it employs only one type of operation) but suffers from the fact that it involves relatively higher IL losses (due to carryover as, unlike in a membrane-based operation such as PV, there is no physical barrier). Furthermore, to accomplish relatively high levels of dehydration (≥90 wt% IL, which is necessary in the case of some ILs such as [C_2_C_1_Im][OAc]), relatively higher temperatures (≥150 °C) are required in the case of the VD configuration, and/or prolonged operation times that could potentially lead to further IL losses due to thermal degradation. Given that biorefineries—like any other manufacturing facility—are expected to be in operation on a nearly continuous basis throughout the lifespan of the facility (typically 30 years), the cumulative IL losses can be significant and could impact performance unless make-up IL is supplied that would incur additional costs. The lost IL ends up in the aqueous streams, thereby incurring additional wastewater treatment costs. To understand the economic impact of the IL losses in the VD configuration, three different scenarios are constructed with varying IL recoveries (98, 99, 99.9%), whereas the IL recovery in the PV system is kept constant at 99.9% (which is feasible due to the lower temperature operation as well as the existence of physical barrier that is impermeable to [C_2_C_1_Im][OAc], as demonstrated in this study). Given the uncertainty with IL prices, two different pricing schemes are considered by varying price between $10 and $2 kg^−1^, which is considered one of the lower price limits for certain ILs [42].

For all the scenarios described above, the economic potential is evaluated by computing minimum ethanol selling price (MESP) through a detailed cash flow analysis. The relative economic impact VD (vs PV) is measured by ∆ (MESP), which is defined as the MESPVD–MESPPV. Subsequently, a positive difference indicates that PV is more economical and vice versa. The resulting ∆ (MESP) values are shown in Fig. 7b, and it is evident that the VD is likely to be more expensive in the scenarios with relatively lower IL recoveries (i.e., 99 or 98 wt%) regardless of whether the IL price is high ($10 kg^−1^) or low ($2 kg^−1^). In the context of thermally sensitive ILs, regardless of IL price, PV is a more advisable configuration. PV is particularly attractive in the case of high IL price ($10 kg^−1^) as the ∆ (MESP) itself is prohibitively expensive (varied between $6 and $12 gal^−1^) rendering VD configuration uneconomical. This is mostly due to the significantly high IL make-up costs in the case of more expensive ILs. In the best-case scenarios studied with highest IL recoveries (~99.9 wt%)—i.e., if the IL recovery in VD is comparable to that of PV—the economic advantage of PV diminishes. It is, however, important to note that given the long lifespan of these manufacturing plants (30 years or more), it may be less likely to attain such high recoveries (≥99.9 wt%) with VD alone especially if the ILs were to be dehydrated to ≥90 wt%. This is particularly challenging in the case of thermally sensitive ILs, and emphasizes the promising role of PV in the context of IL recovery and recycles. We also recognize that the economic potential of PV would be subjected to the factors such as membrane costs that are uncertain at this point. Therefore, to understand the potential impact of PV membrane costs on the overall economics, we conducted additional sensitivity analysis by varying PV membrane purchase costs by ±50%. Subsequent change in MESP was found to be fairly small (within ±3%). Thus, the MESP appears to be less sensitive to the PV membrane costs. Likewise, the sensitivity analysis based on ±50 variation in VD cost resulted in rather small variation in MESPs (i.e., around 2–6% variation depending on the IL price and recovery). This insensitivity can be attributed to the fact that there are other significant cost drivers—i.e., energy intensity of IL dehydration as it involved dehydration from dilute conditions (<20 wt% IL) to near dry conditions (>90 wt%) regardless of the technology choice (i.e., PV or VD). Subsequently, the MESP remained around $7 gal^−1^ or more in the scenarios investigated (Additional file 1: Figure S9). Therefore, although our study has successfully demonstrated the use of PV for high levels of IL dehydration, further upstream improvements are necessary (e.g., minimize water usage, therefore reducing the amount of water to be dehydrated subsequently) to improve overall energy efficiency of the process. Such advancements are possible with biocompatible ILs [7, 43]. Combing the merits of PV and biocompatible ILs, it is possible to design biorefineries that are efficient in terms of IL recovery as well as energy usage. In this context, as shown in this study, since PV can be used over a wide concentration regime—from dilute (i.e., ~20 wt% IL) to near-complete dry condition (i.e.,>99 wt% IL)—PV could potentially broaden the types and number of ILs that can be used in biorefinery applications.

## Conclusions

The present research aimed to develop and demonstrate an efficient and robust technology for the dehydration, recovery, and reuse of [C_2_C_1_Im][OAc] after lignocellulosic biomass processing. We evaluated pervaporation (PV) in place of conventional distillation to recover the [C_2_C_1_Im][OAc] after pretreatment. Compared to vacuum distillation and electrodialysis, we found that the [C_2_C_1_Im][OAc] loss was kept within 0.1 wt% (i.e., >99.9 wt% IL recovery) using PV, and near-complete dehydration of IL (i.e., >99 wt% IL) was achieved with a maximum water flux of 42.8 kg h^−1^ m^−2^. Overall, the separation was found to very effective with separation factors of ~1500. The recovered [C_2_C_1_Im][OAc] was reused five times without significant changes in chemical structure and pretreatment efficiency. In addition, the long-term stability of the PV membrane has been demonstrated over 60 dehydration cycles using the same [C_2_C_1_Im][OAc]-water mixture. A preliminary technoeconomic analysis highlights the advantage of PV in conjunction with vacuum distillation, as it could potentially minimize IL losses thereby improving overall economics. PV integrates evaporation with a permeation membrane and has the potential to meet the needs for both high selectivity and low IL loss. More efforts are still needed to improve the permeate flux, selectivity, and stability of the membranes in a more complex separations and scale-up applications with significant prospects in fuels and chemical industries.

## Methods

### Materials

The two feedstocks included in this study were switchgrass (*Panicum virgatum*) and eucalyptus (*Eucalyptus cinerea*). The origin, the harvesting, and detailed grinding and drying parameters of the feedstocks are described elsewhere [44]. After the grinding and drying steps, equal quantities of each feedstock (1:1 on dry weight basis) were blended and mixed for biomass pretreatment. [C_2_C_1_Im][OAc] with a purity of >99% was purchased from BASF (Florham Park, NJ, USA) and used as received. Cellulase (Cellic^®^ CTec2; Batch# VCN10001, protein content 188 mg mL^−1^) and hemicellulase (Cellic^®^ HTec2; Batch# VHN00001, protein content 180 mg ml^−1^) enzyme mixtures were received as gifts from Novozymes NA (Franklinton, NC, USA), and mixed with the volume ratio of 9:1 before use. Polysulfone ultrafiltration ER membranes (30 kD, 47 mm, YMERSP475) were purchased from Sterlitech Corporation.

### Biomass pretreatment

As an example, 0.5 g of switchgrass and 0.5 g of eucalyptus were mixed with 8.1 g of [C_2_C_1_ m][OAc] and 0.9 g of water to give a 10 wt% biomass loading. Pretreatment runs were carried out at 160 °C for 1 h with constant stirring at 120 rpm by an 80-mm-diameter polytetrafluoroethylene anchor-type impeller, powered by a Heidolph RZR 2052 mechanical stirrer (Heidolph Instruments GmbH & Co. KG, Schwabach, Germany). Duplicate runs were performed for each IL pretreatment of mixed feedstocks. After pretreatment, the slurry was washed five times with DI water to remove the residual [C_2_C_1_im][OAc]. An aliquot of recovered solid was lyophilized in a FreeZone^®^ Freeze Dry System (Labconco, MO, USA) and used for composition analysis. All the water-washed streams were collected and used as the raw feed solution for pervaporation, in which the [C_2_C_1_Im][OAc] concentration was controlled at ~20 wt%.

### Pervaporation operation

In a typical pervaporation process, 50 g 20 wt% IL feed solution was added to a 125 mL stainless steel vessel. The Dewar was filled about 3/4-way of liquid N_2_ and then was connected with insulation foam cap under condenser and slowly immerse condenser in coolant. After connecting thermocouple to heater/temperature controller box and installing insulation block, the vessel was heated up to a desired operating temperature with stirring and N2 sweeping (a flow rate of 100 mL min^−1^). Conductivity measurements were performed at room temperature (20 °C) in triplicate using a S230 SevenCompact conductivity meter (Mettler-Toledo, LLC) with an accuracy of ±0.5%. A thorough water wash of membranes in between tests is required using the same apparatus at 100 °C for 1–2 h.

### Enzymatic hydrolysis

Enzymatic saccharification of pretreated and untreated biomass samples were carried out in duplicates based on the NREL laboratory analytical protocol 9 ‘Enzymatic Saccharification of Lignocellulosic Biomass’ [45]. The citrate buffer (final molarity 50 mM), enzymes, and DI water were mixed with the recovered solids after pretreatment to achieve a final solids loading of around 10 wt%. A 20 mg protein g^−1^ solid of enzyme loading was used unless otherwise specified. The supernatant collected during 72 h of hydrolysis was analyzed by HPLC as previously described in literature [19]. Glucose and xylose yields were calculated based on the theoretical glucose and xylose yields as determined by compositional analysis of the recovered biomass after pretreatment. After 72 h of hydrolysis, the remaining solids were collected by centrifugation and washed with an excess volume of DI water to remove residual sugars. The solids were then lyophilized and analyzed for acid-insoluble lignin, glucan, and xylan compositions.

### Characterizations of ultrafiltration membrane

#### Scanning electron microscopy (SEM)

SEM images were taken for both new and 5th used UF membranes using a Hitachi S-5000 microscope. Prior to acquiring images, the samples were mounted with double-sided carbon tape on precut brass sample stubs and sputter coated with approximately 30 Angstrom of Au/Pd. The representative images of membranes in this work were acquired with a 10 kV accelerating voltage and scanned with 50,000 magnification.

#### Fourier transform infrared (FTIR) Spectroscopy

All the samples were cleaned with DI-water under ultrasonic conditions and dried at 45 °C under vacuum for 2 days. FT-IR spectra were collected in the Mid-IR region (2000–600 cm^−1^) with 4 cm^−1^ resolution using Bruker Optics Vertex system (Billerica, MA, USA) with a built-in diamond–germanium ATR single reflection crystal. Air was used as background for all the samples. A set of 96 scans was collected for each sample. All the samples were baseline corrected and vector-normalized using OPUS software from Bruker Optics.

#### Technoeconomic analysis (TEA)

The model of biorefinery process in Fig. 7a was built up in SuperPro designer (v8.5). It was assumed that the pretreatment was carried out at 20 wt% biomass loading (as higher loadings are generally preferred to improve overall process economics) and a water loading (i.e., mass ratio between total amount of fresh water used and dry biomass present) of 20 in the subsequent water-wash step. All the IL is recovered into the aqueous stream and the impact of any residual IL present in the pretreated biomass on the hydrolysis and fermentation is assumed to be negligible. Downstream hydrolysis was conducted at 20% solid loading with an enzyme loading of 20 mg g^−1^ solid (i.e., the total solids recovered in the washing step after pretreatment). Fermentable sugars in the hydrolysate were assumed to be co-fermented to produce ethanol that was recovered from the broth using distillation columns in the product recovery section. Key process parameters used in this TEA are provided in Additional file 1: Table S1. While several parameters (e.g., operating temperature of pretreatment and subsequent IL dehydration, enzyme loading) are based on the experimental demonstration in this study, three important parameters (i.e., solids loading during pretreatment and hydrolysis, sugar and ethanol yields) are based on projected performance of a target Nth plant scenario that can be realized with continued developments. Cost data for most of the equipment, and other production costs (i.e., raw materials, labor, and energy), and assumptions for economic analysis were taken from previous studies [5, 40]. Since this is assumed to reflect an industrial scale facility, appropriately sized (large) vessels are assumed to be available to perform key operations—for instance, based on NREL study [40], vessels with a volume of one million gal are used to perform fermentation. Subsequently, based on amount of material processed and processing times, the number of vessels to be utilized in parallel (to satisfy the total volume requirement) was determined. Costs of major equipment are computed based on the equipment cost data and scaling factors (mostly varied in the range of 0.6–0.8) given in NREL study [40]. With perceived advances and based on a large-scale production of PV membranes, we assumed membrane purchase cost of $8 ft^−2^ in this study (in addition, given the uncertainty with membrane cost, a sensitivity analysis is conducted by varying the membrane cost by ±50%). In addition, the cost of industrial scale evaporator with an effective surface area of around 814 m^2^ was estimated to be around MM $2.04 and, to account for any uncertainty, a sensitivity analysis is conducted with ±50%. Variation in line with these studies, minimum ethanol selling price (MESP) was used as a key economic performance indicator and was computed through a detailed cash flow analysis over a 30 year project life. The MESP was equivalent to the selling price of ethanol from the cash flow analysis at 10% internal rate of return. Base year for economic analysis in current study is 2014.

In order to understand the economic impact of IL recovery, pretreatment section was modeled in detail and includes pretreatment, water-wash step, ultrafiltration (to remove insoluble solids), and IL concentration/drying operations (i.e., PV and/or VD) to recover/recycle IL from aqueous IL solution (about 85 wt% water). To understand the relative economic merit of PV and VD, two different configurations are studied (Additional file 1: Figure S7): (1) PV/hybrid configuration, (2) VD configuration. In both cases, it is assumed that the IL needs to be dehydrated to around 10 wt% water. The PV/hybrid configuration involved an initial feed concentration (from around 85 to 60 wt% water) followed by a PV membrane to further dry IL (from 60 to 10 wt % water) so that it can then be readily recycled to pretreatment reactor. A multi-effect vacuum distillation (MEVD) system is considered for initial feed concentration step. An average flux of 0.5 kg m^−2^ h^−1^) is assumed for the PV system. In the VD configuration, only MEVD is employed to concentrate and dry IL (from 85 to 10 wt% water). Since the VD configuration needs to dry IL to high IL concentration (≥90 wt% IL), typically, it requires relatively higher temperatures (≥150 °C) and/or extended operation times. Subsequently, medium pressure (9.5 bar) steam is utilized in the VD configuration (where as low pressure steam is utilized in the PV configuration). Furthermore, a backward feeding strategy employed in the VD configuration to ensure that the last effect with higher IL concentration is maintained at higher temperatures. Subsequently, the concentrated IL stream in the VD configuration is used to partly pre-heat the aqueous IL feed stream.

## Additional file

**Additional file 1: Figure S1.** Relationship of conductivity and [C_2_C_1_Im][OAc] concentration. **Figure S2.** LC results from LC/MS on multiple drying cycles of [C_2_C_1_Im][OAc]/H_2_O. **Figure S3.** Drying [C_2_C_1_Im][OAc]-water. (a) Trials using 5 h, 100 °C conditions, no proper membrane cleaning; (b) 100 °C, 50 wt% initial water content. **Figure S4.** Ultra-filtration (UF) treatment of ionic liquid feed solution (a), and characterizations of UF membrane before and after use (b, SEM; c, FTIR). **Figure S5.** Ionic liquid and water streams of the optimized biomass pretreatment process (S represents the solid stream, and L represents the liquid stream). **Figure S6.** 1H-NMR spectrums of IL (a: before; b: after 5th reuse) and permeate (c). **Figure S7.** Home-built Bench-top electrodialysis (ED) apparatus. **Figure S8.** Two IL recovery configurations studied in TEA-PV/hybrid configuration (top) and VD configuration (bottom). **Table S1.** Key process and cost data used in the TEA.

## Abbreviations

IL: ionic liquid
[C_2_C_1_Im][OAc]: 1-ethyl-3-methylimidazolium acetate
PV: pervaporation
[C_4_C_1_Im]Cl: 1-butyl-3-methylimidazolium chloride
ED: electrodialysis
RO: reverse osmosis
Barrer: a unit of gas permeability used in the membrane technology, which is named after Richard Barrer. 1 Barrer is equivalent to 3.34 × 10^−16^ mol Pa^−1^ s^−1^ m^−1^
UF: ultrafiltration
SEM: scanning electron microscopy
and FTIR: Fourier transform infrared
1H-NMR: proton nuclear magnetic resonance
DI water: deionizing Water
VD: vacuum distillation
TEA: technoeconomic analysis
MESP: minimum ethanol selling price
MEVD: multi-effect vacuum distillation
