# Supplementary material for: Efficient dehydration and recovery of ionic liquid after lignocellulosic processing using pervaporation
Source: Biotechnol Biofuels. 2017 Jun 15;10:154. doi: 10.1186/s13068-017-0842-9 (PMC5472906; doi:10.1186/s13068-017-0842-9)
Supplement: Supplementary file 1 — Additional file 1: Figure S1. Relationship of conductivity and [C2C1Im][OAc] concentration. Figure S2. LC results from LC/MS on multiple drying cycles of [C2C1Im][OAc]/H2O. Figure S3. Drying [C2C1Im][OAc]-water. (a) Trials using 5 h, 100 °C conditions, no proper membrane cleaning; (b) 100 °C, 50 wt% initial water content. Figure S4. Ultra-filtration (UF) treatment of ionic liquid feed solution (a), and characterizations of UF membrane before and after use (b, SEM; c, FTIR). Figure S5. Ionic liquid and water streams of the optimized biomass pretreatment process (S represents the solid stream, and L represents the liquid stream). Figure S6. 1H-NMR spectrums of IL (a: before; b: after 5th reuse) and permeate (c). Figure S7. Home-built Bench-top electrodialysis (ED) apparatus. Figure S8. Two IL recovery configurations studied in TEA-PV/hybrid configuration (top) and VD configuration (bottom). Table S1. Key process and cost data used in the TEA. [file 13068_2017_842_MOESM1_ESM.docx]

**Electronic Supporting Information**

Efficient Dehydration and Recovery of Ionic Liquid after Lignocellulosic Processing Using Pervaporation

Jian Sun*^a,b^*, Jian Shi*^a,b,c^*, N. V. S. N. Murthy Konda *^a^*^,^*^d^*, Dan Campos^e^, Dajiang Liu*^a,b^*, Stuart Nemser*^e^*, Julia Shamshina*^f,g,h^*, Tanmoy Dutta*^a,b^*, Paula Berton*^f,g^*, Gabriela Gurau*^f,h^*, Robin D. Rogers*^f,g^*, Blake A Simmons*^a,d^*, Seema Singh*^a,b*^*

*^a^* Deconstruction Division, Joint BioEnergy Institute, Emeryville, CA 94608, USA.

*^b^* Biological and Engineering Sciences Center, Sandia National Laboratories, Livermore, CA 94551, USA.

*^c^*  Biosystems and Agricultural Engineering, University of Kentucky, Lexington, KY 40546, USA.

*^d^*  Biological Systems and Engineering Division, Lawrence Berkeley National Laboratory, Berkeley, CA 94720, USA.

*^e^* Compact Membrane Systems Inc, Newport, DE 19804, USA.

^f^ Department of Chemistry, The University of Alabama, Tuscaloosa, AL 35487, USA.

*^g^* Department of Chemistry, McGill University, 801 Sherbrooke St. West, Montreal, QC H3A 0B8, Canada.

*^h^* 525 Solutions, Inc., Tuscaloosa, AL 35401 USA.

* Corresponding author: E-mail: [seesing@sandia.gov](mailto:seesing@sandia.gov)

**Figure S1.** Relationship of conductivity and [C_2_C_1_Im][OAc] concentration


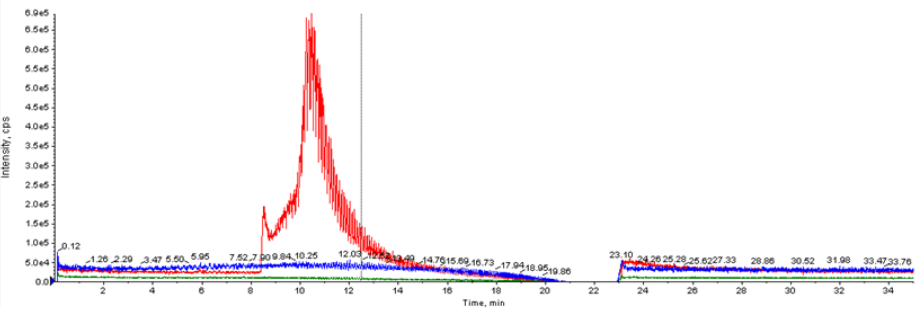

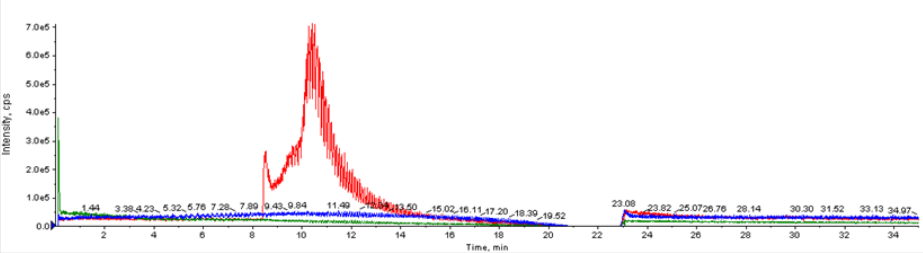


New 50% [C_2_mim][OAc]-H_2_O

Used 50% [C_2_mim][OAc]-H_2_O

40+ large scale pervaporation cycles in CMS

**Figure S2.** LC results from LC/MS on multiple drying cycles of [C_2_C_1_Im][OAc]/H_2_O.

**
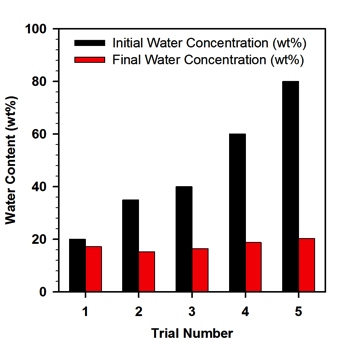
**

**a**

**
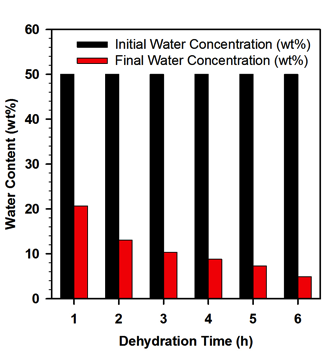
**

**b**

**Figure S3.** Drying [C_2_C_1_Im][OAc]-water. (a) Trials using 5 h, 100 °C conditions, no proper membrane cleaning; (b) 100 °C, 50 wt% initial water content.

**a**


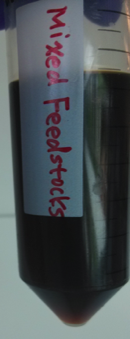

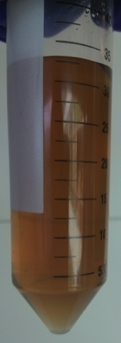


**Before UF**

**After UF**


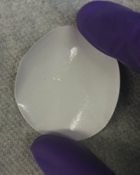

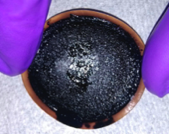


**UF membrane/30kD**

**Soluble lignin**

**IL/H_2_O**

**b**

**
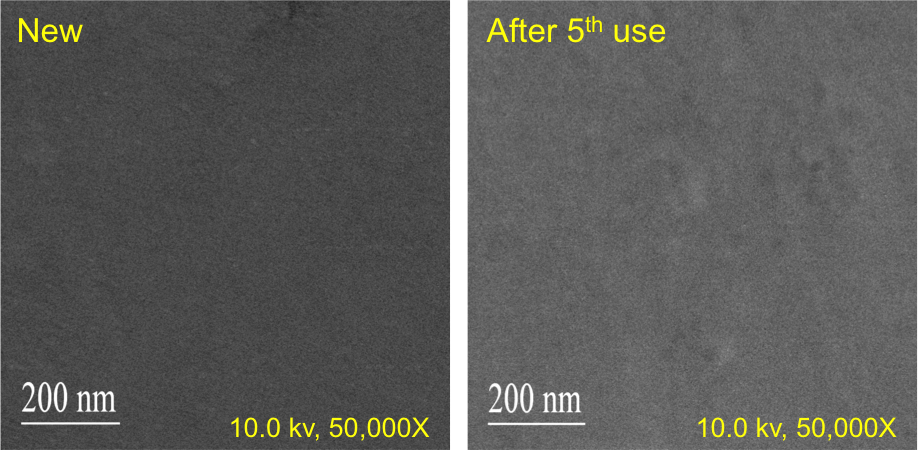
**

**c**

**Figure S4.** Ultra-filtration (UF) treatment of ionic liquid feed solution (a), and characterizations of UF membrane before and after use (b, SEM; c, FTIR)

**Figure S5.** Ionic liquid and water streams of the optimized biomass pretreatment process (**S** represents the solid stream, and **L** represents the liquid stream).

**c**

**b**

**a**


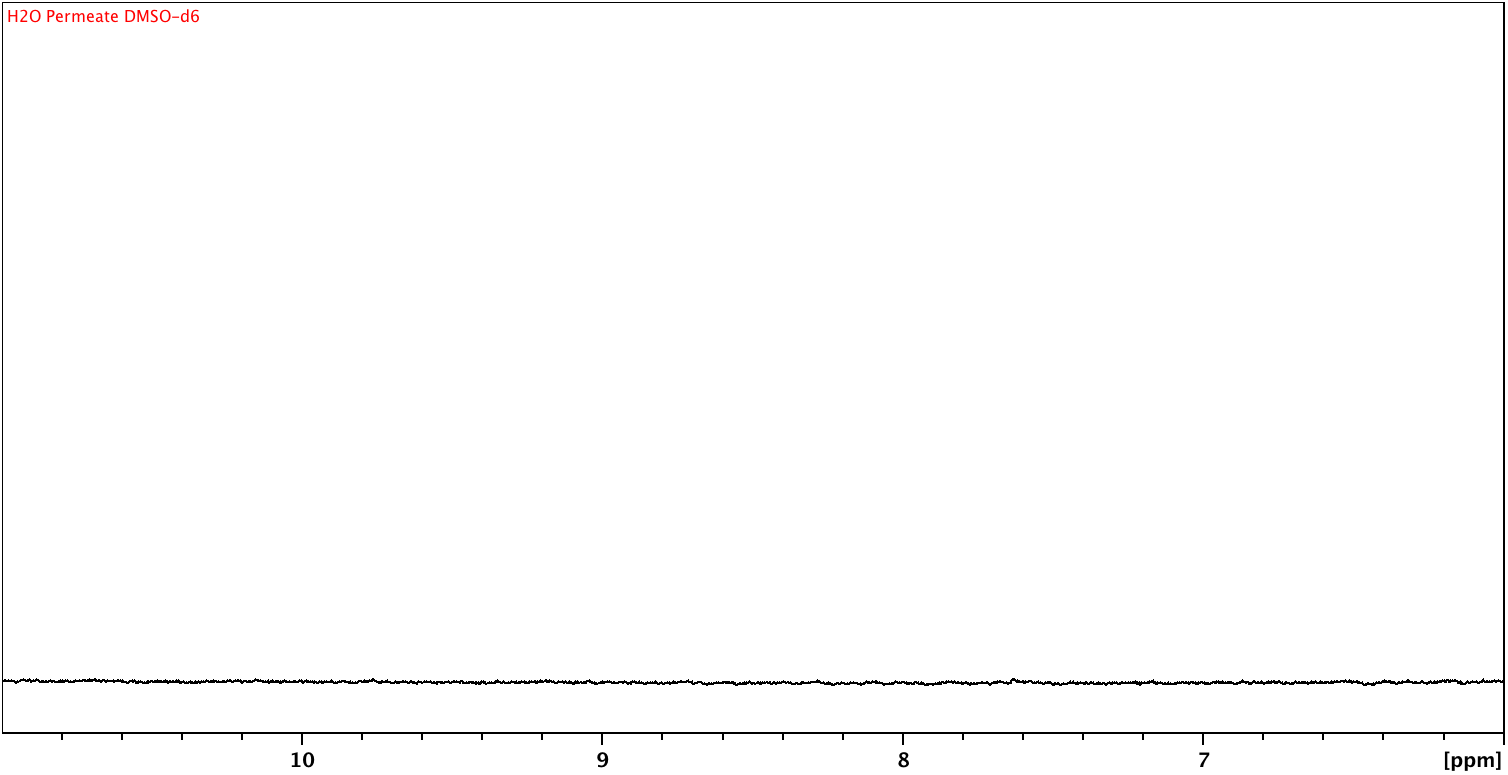

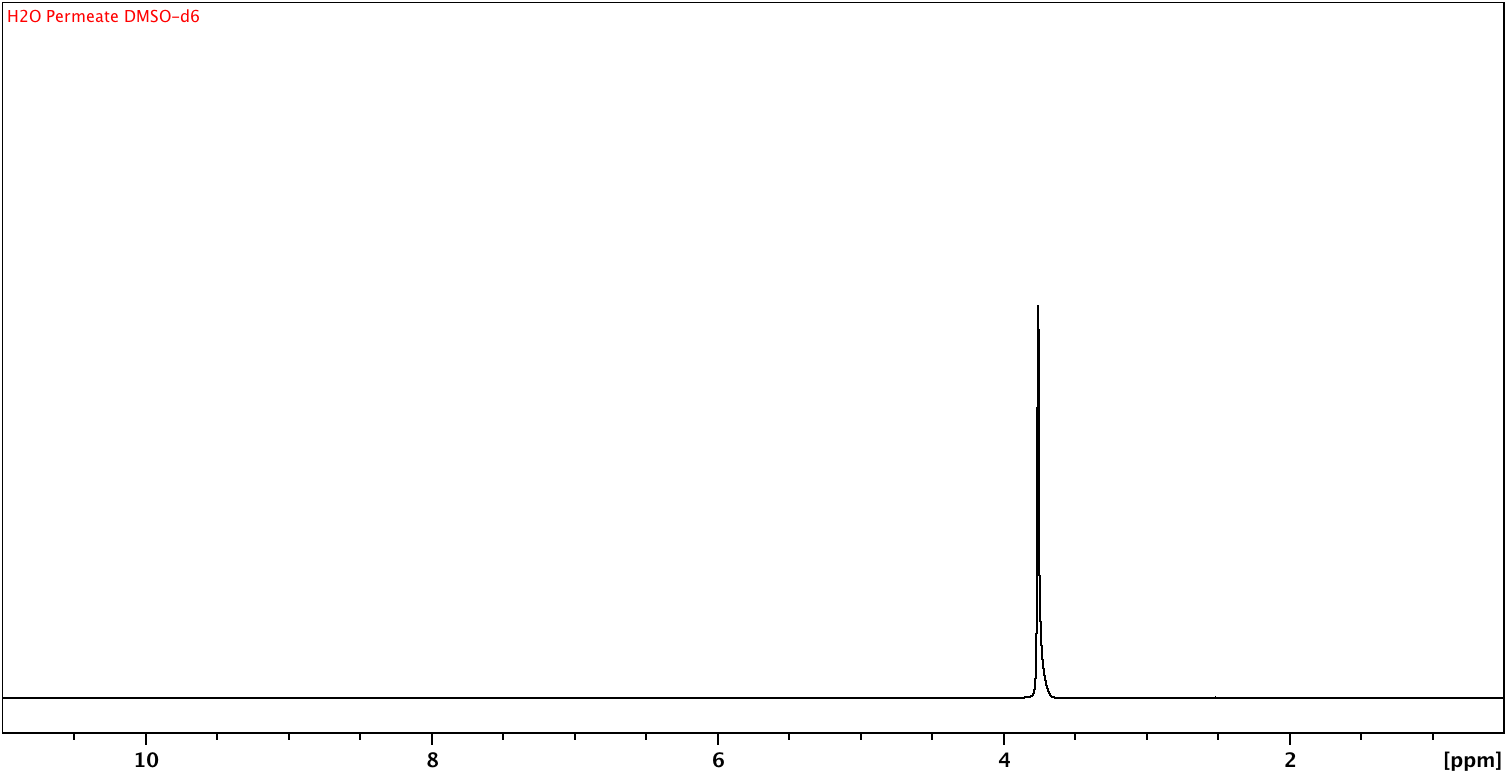


**H_2_O**

**Figure S6.** ^1^H**-**NMR spectrums of IL (a: before; b: after 5^th^ reuse) and permeate (c).


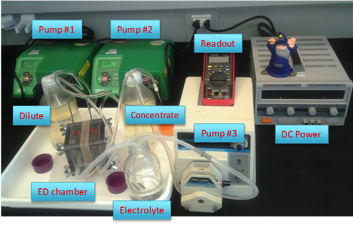


**Figure S7.** Home-built Bench-top electrodialysis (ED) apparatus. The ED chamber is made of 6 pairs of anion exchange membranes (AEM, item# AMI-7001S) and cation exchange membranes (CEM, item# CMI-7000S, both membranes are purchased from Membrane International, Ringwood, NJ). The membranes are spaced by silicone gasket (1mm thickness) with precut openings to create a counter-flow mode for the dilute and concentrate streams. At time 0, both reservoirs (supplying the dilute and concentrate streams) contain 10 wt% [C_2_C_1_Im][OAc] in water. The [C_2_C_1_Im][OAc] concentrations were monitored by measuring the conductivity in both reservoirs during 5 h ED separation (at flow rate, 6 mL/min; voltage, 20 volts). The final concentration of [C_2_C_1_Im][OAc] in one reservoir was 0.7 wt% (dilute stream), while the other reservoir was 45 wt% (concentrate stream) and both stayed unchanged after ~4.5 h.

**Figure S8.** Two IL recovery configurations studied in TEA-PV/hybrid configuration (top) and VD configuration (bottom).

**Figure S9.** Estimated MESP values for the scenarios studied (includes three VD scenarios with varying IL recoveries from 98% to 99.9%, and PV/hybrid scenario with 99.9% IL recovery.

Table S1. Key process and cost data used in the TEA.

| **Feedstock mixture (w/w)** | | Eucalyptus & Switchgrass (1:1) |
| --- | --- | --- |
|  | Feedstock composition (wt%, dry basis) |  |
|  | Glucan | 32.2 |
|  | Xylan | 16.7 |
|  | Lignin | 25.0 |
|  | Others | 26.1 |
|  | Moisture content in the delivered feedstock at plant-gate (wt%) | 20 |
|  | Biomass processed (dry) (MT/day) | 2000 |
|  | Biomass price (delivered at plant-gate) ($/dry ton) | 80 |
| **Pretreatment (inc. water-washing, IL) recovery/recycle)** | |  |
|  | IL used | [C_2_C_1_Im][OAc] |
|  | IL purity (wt% of IL in aqueous IL solution [IL:H_2_O]) | 90 |
|  | Biomass loading (wt% dry biomass during pretreatment) | 20 |
|  | Operating temperature (°C) | 160 |
|  | Operating time (h) | 1~3 |
|  | Water loading (wt%)(mass ratio between total amount of fresh water used and pretreated biomass in water-wash step) | 20 |
|  | Pervaporation operating temperature (°C) | 80~100 |
| **Hydrolysis** | |  |
|  | Enzyme loading (mg/g solids in hydrolyser feed) | 20 |
|  | Solids loading (wt% of solids during hydrolysis) | 20 |
|  | Operating temperature (°C) | 50 |
|  | Operating time (hr) | 72 |
|  | Enzyme price ($/kg protein) | 4.29^a^ |
|  | Glucan-to-glucose conversion (%) | 98 |
|  | Xylan-to-xylose conversion (%) | 80 |
| **Fermentation** | |  |
|  | Co-utilization of glucose and xylose | Yes |
|  | Glucose-to-ethanol conversion (%) | 90 |
|  | Xylose-to-ethanol conversion (%) | 80 |
| ^a^ Effective price of enzyme estimated based on NREL study (See ref. 24) | | |
